# Combined Femtosecond Laser-Assisted Cataract Surgery and 27-Gauge Transconjunctival Sutureless Vitrectomy

**DOI:** 10.1155/2020/7651941

**Published:** 2020-03-23

**Authors:** Sami Yılmaz, Remzi Avcı, Ayşegül Mavi Yıldız

**Affiliations:** Bursa Retina Eye Hospital, Bursa, Turkey

## Abstract

**Purpose:**

To report the outcomes of combined surgery using femtosecond laser-assisted cataract surgery (FLACS) and sutureless 27-gauge pars plana vitrectomy with intravitreal tamponade.

**Methods:**

This retrospective clinical study involved 23 eyes of 23 patients on whom combined vitreoretinal surgery was performed. Patients were initially given the femtosecond laser treatment that was performed after selection of capsulotomy and lens fragmentation patterns. The capsulotomy diameter was chosen as 4.9 mm in all patients. After the femtosecond laser, the sutureless phacovitrectomy procedure was performed. At the end of surgery, perfluoropropane or sterile air tamponade was applied.

**Results:**

The mean age of patients was 66.43 ± 7.61 (range, 54–83) years. Fifteen patients were females (65.2%). The mean follow-up was 16.09 ± 4.71 (range, 9–25) months. The most common surgical indication was epiretinal membrane (65.3%). The mean preoperative best-corrected visual acuity (BCVA) was 0.71 ± 0.44 (range, 1.7–0.3) logMAR, and the mean postoperative BCVA at 6 months was 0.16 ± 0.14 (range, 0.4–0) logMAR (*p* < 0.001). The mean target sphere refractive error was −0.24 ± 0.16 (range, −0.50–0.11) D, and the mean postoperative spherical equivalent refractive error was −0.14 ± 0.39 (range, −1.00–0.50) D at 6 months (*p*=0.196). All intraocular lenses (IOLs) remained well centered in the capsular bag during surgery and follow-up. There was no iris capture, posterior synechiae, capsular opacification, or pseudophakic cystoid macular edema. The only complication related to femtosecond laser was two cases of subconjunctival haemorrhage related with suction.

**Conclusions:**

FLACS is a safe and effective technique providing the advantage of repeatable, precise capsulorhexis shape and size to achieve a well-centered and stable IOL postoperatively. These advantages can certainly improve the results of vitrectomy, especially in gas-filled eyes. FLACS and 27-gauge sutureless combined surgery may be a future trend in appropriate cases.

## 1. Introduction

Vitreoretinal disease with coexisting cataract is common in elderly patients. The initiation of phacoemulsification in the 1990s made combined cataract and vitrectomy surgery a practical and safe procedure [1]. Phacovitrectomy provides faster postoperative visual rehabilitation in addition to clear retinal visualization during vitreous surgery [2, 3].

Femtosecond laser-assisted cataract surgery (FLACS) is a recent innovation in phacovitrectomy. It offers several advantages over conventional phacoemulsification, such as a decrease in the energy of the phacoemulsification during cataract surgery and precise and predictable continuous curvilinear capsulorhexis, which affords improved intraocular lens (IOL) centralization and stability [4]. Intravitreal tamponades have an important impact on the position of the IOL in phacovitrectomy, such as tilt and decentralisation [5, 6]. We report the results of combined surgery using FLACS and sutureless 27-gauge pars plana vitrectomy with intravitreal tamponade.

## 2. Materials and Methods

A retrospective, observational, and consecutive review of the clinical records of patients diagnosed with coexisting retinal pathologies and cataract who underwent combined FLACS and sutureless 27-gauge vitreoretinal surgery between May 2017 and February 2019 at Bursa Retina Eye Hospital was performed. All subjects provided informed written consent and procedures were performed in accordance with the Declaration of Helsinki. The inclusion criteria included the presence of coexisting cataract and vitreoretinal pathology treated with concurrent FLACS and 27-gauge vitrectomy with a minimum follow-up of 6 months. The exclusion criteria are listed in Table 1.

The examinations included preoperative best-corrected visual acuity (BCVA), target sphere refractive errors (Nidek Optical Biometer-AL Scan, Nidek Co., Ltd., Japan), intraocular pressure (IOP), slit-lamp biomicroscopy, fundus examination, color fundus photography, spectral domain optical coherence tomography (SD-OCT), type of cataract and vitreoretinal disease, age, and sex. Postoperative follow-up was performed at day-1, at week-1, and at month-1, month-3, and month-6. On all visits, IOP, refraction, and postoperative complications were recorded. The BCVA was also assessed at postoperative month-1, month-3, and month-6.

### 2.1. Surgical Technique

**Figure 1:**
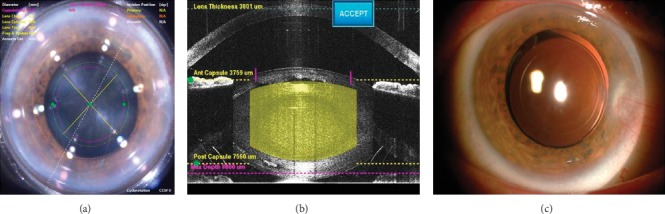
The capsulotomy diameter was chosen as 4.9 mm (a). The lens anterior and posterior offsets were set at 500 *μ*m and 800 *μ*m (b). Anterior segment photography of an eye following dilation of the pupil at postoperative 6 months. The IOL is well centered in the capsular bag (c).

Patients were initially placed in the femtosecond laser operating suite. Femtosecond laser (Alcon LenSx Inc., Aliso Viejo, Calif., USA) treatment was performed in the operating suite under topical anesthesia (proparacaine hydrochloride 0.5%). Once the docking procedure had been achieved with a disposable patient interface, the laser treatment was carried out after selection of capsulotomy and lens fragmentation patterns. The capsulotomy diameter was chosen as 4.9 mm (Figure 1(a), as an example), with capsule delta up and down of 250 *µ*m and 350 *µ*m, respectively. The method of lens fragmentation was used for chopping. The lens anterior and posterior offsets were set at 500 *μ*m and 800 *μ*m (Figure 1(b), as an example).

After femtosecond laser, patients were taken to another operating room for the sutureless phacovitrectomy procedure. Retrobulbar block anesthesia (lidocaine hydrochloride 2%) was used. After preparing the surgical field, the surgeon (R.A.) performed 2.2 mm clear corneal incision manually approximately 0.2 mm anterior to the edge of the limbal vessels. The axis of the main incision was approximately 135 degrees (superotemporal in the right eyes and superonasal in the left eyes) in all eyes. Subsequently, sodium hyaluronate 1% was instilled into the anterior chamber. The anterior capsule was then extracted with forceps. Hydrodissection, phacoaspiration, and irrigation-aspiration of the lens masses were performed (Centurion Vision System, Alcon Laboratories Inc., Fort Worth, TX, USA). Finally, monofocal Alcon AcrySof SN60WF, 6 mm diameter intraocular lens implantation was performed with balanced salt solution irrigation (hydroimplantation). The incision was self-sealing, and mild edema was induced around the incision site by hydration.

The same surgeon created three transconjunctival pars plana ports at inferotemporal, superotemporal, and superonasal quadrants 3.5 mm from the limbus using 27-gauge trocars (D.O.R.C. International and Alcon Laboratories). Vitrectomy was performed using the 27-gauge vitrectomy system of the DORC (Dutch Ophthalmic Research Center, Zuidland, Netherlands) and Zeiss microscope with EIBOS 2 (Haag Streit, USA) attachment for noncontact fundus viewing. For all patients, a near-complete vitrectomy, including vitreous base shaving, epiretinal membrane (ERM) and internal limiting membrane (ILM) peeling, laser endo-photocoagulation, inspection of the peripheral retina for tears, and partial fluid-air exchange for prevention of leaking sclerotomies or a non-expansile mixture of C3F8 gas tamponade was performed depending on the type of vitreoretinal pathology. On completion of the procedure, the microcannulas were removed and the eye was inspected for any signs of wound leak and the position of the IOL was reexamined.

### 2.2. Postoperative Course

Patches were applied to all eyes until the first postoperative day visit. Postoperative medical treatment included one drop each of a fourth generation fluoroquinolone (moxifloxacin hydrochloride, Vigamox, Alcon) and 0.5% tropicamide (Tropamid, Bilim) four times a day and 0.1% dexamethasone ophthalmic suspension (Maxidex, Alcon) eight times a day for a week. Steroid drops were then gradually reduced in frequency over the following 3 weeks. In addition, if needed, topical glaucoma medication was included in the treatment regimen. Patients were instructed to spend most of the day in prone position for 7 days with gas tamponades (for full-thickness macular hole and rhegmatogenous retinal detachment) and 3 days with air tamponades (for ERM, vitreous haemorrhages, proliferative diabetic retinopathy, and vitreomacular traction).

Postoperative follow-up was performed on the first day, at 1 week, at 1 month, at 2 months, and at 6 months. On visits, postoperative spherical equivalent refractive errors, BCVA, IOP, slit-lamp biomicroscopy, and fundus examination were included.

### 2.3. Statistical Analysis

BCVA was measured using the Snellen chart and converted to the logarithm of the minimum angle of resolution (logMAR) scale for statistical analysis. Descriptive statistics were reported as mean ± standart deviation (SD). Data were compared with the paired *t*-test and repeated measures ANOVA was used for comparing measures of BCVA and IOP during the follow-up period. Bonferroni adjustment was used as the post-hoc test. Postoperative spherical refractive errors were reported to the nearest 0.25 D. All statistical analyses were performed using IBM SPSS Statistics for Windows, version 21.0 (IBM Corp, Armonk, NY). *p* < 0.05 was considered to be statistically significant.

## 3. Results

The mean age of the study patients was 66.43 ± 7.61 (range, 54–83) years. Fifteen patients were females (65.2%). The mean follow-up was 16.09 ± 4.71 (range, 9–25) months.

### 3.1. Indications for Surgery

Indications for vitreoretinal surgery were visually significant ERM in 15 (65.3%) eyes. Four (17.5%) full-thickness macular holes and one each of proliferative diabetic retinopathy (4.3%), vitreous haemorrhages (4.3%), rhegmatogenous retinal detachment (4.3%), and vitreous macular traction (4.3%) were also included. Eye lens types were categorized as cortical cataract (*n* = 10, 43.5%), nuclear cataract (*n* = 7, 30.5%), nuclear sclerosis (*n* = 2, 8.7%), posterior subcapsular cataract (*n* = 2, 8.7%), both nuclear and cortical cataract (*n* = 1, 4.3%), or both nuclear and posterior subcapsular cataract (*n* = 1, 4.3%). Cortical cataracts were detected most often.

### 3.2. Surgical Outcomes

The surgery was successfully completed in all patients without any major complications. Postoperatively, all cases had clear corneas, well-centered IOLs, and attached retina. Mild subconjunctival hemorrhage was detected in two subjects due to the application of the docking system and suction. No dislocation of the intraocular lens into the anterior chamber was observed, despite the scleral indentation without corneal sutures, in any patient. In all patients, the monofocal foldable IOL remained stable and their corneas were clear during the vitreoretinal surgery.

The mean time of the phacoemulsification and intraocular lens implantation was 14.65 ± 0.93 (range, 13–16) minutes. The mean total surgical time was 50.31 ± 9.01 (range, 40–75) minutes. The mean preoperative BCVA was 0.71 ± 0.44 (range, 1.7–0.3) logMAR. The mean postoperative BCVA was 0.23 ± 0.11 (range, 0.4–0), 0.17 ± 0.13 (range, 0.4–0), and 0.16 ± 0.14 (range, 0.4–0) logMAR at 1 month, 3 months, and 6 months, respectively. The statistical analysis revealed a significant improvement in visual acuity at 1 month (*p*  <  0.001), at 3 months (*p* < 0.001), and at 6 months (*p* < 0.001). The mean preoperative IOP was 15.65 ± 2.95 (range, 11–21) mmHg. The mean postoperative IOP was 18.61 ± 3.04 (range, 10–24), 16.48 ± 3.38 (range, 8–24), 15.83 ± 2.35 (range, 12–20), 15.95 ± 2.69 (range, 10–22), and 15.78 ± 2.79 (range, 11–22) mmHg at 1 day, 1 week, 1 month, 3 months, and 6 months, respectively. The statistical analysis revealed a significantly higher IOP at postoperative day-1 (*p*=0.013). However, none of the eyes reached an IOP above 25 mmHg. At postoperative month-1, two of the patients developed ocular hypertension (an IOP above 25 mmHg) which resolved with topical antiglaucoma medications.

Mean IOL power was 22.24 ± 2.07 (range, 15.5–25) D. The mean target spherical refractive error was −0.24 ± 0.16 (range, −0.50–0.11) D. The mean postoperative spherical equivalent refractive error was −0.14 ± 0.39 (range, −1.00–0.50) D at 6 months. Comparison of preoperative target spherical refractive errors and postoperative spherical equivalent refractive errors at 6 months showed no statistically significant difference (*p*=0.196).

Postoperatively, all patients had clear corneas and attached retina. All IOLs remained well centered in the capsular bag during follow-up (Figure 1(c), as an example). There was no iris capture, posterior synechiae, fibrin reaction, capsular opacification, or pseudophakic cystoid macular edema. Two patients had subconjunctival haemorrhage related with suction.

## 4. Discussion

Cataract formation and progression are an inevitable result of pars plana vitrectomy, and the vast majority of patients on whom PPV is performed will require later cataract surgery [7–9]. The most conspicuous potential advantage of combined surgery is preventing the patient from undergoing a further operation with high costs, anesthesia, and surgical risks. Combined surgery can improve visualization for more precise retinal work, such as epiretinal or internal limiting membrane peeling, and provide a more thorough peripheral shaving of the vitreous base. Of course, there are also disadvantages of combined surgery. These patients tend to have more postoperative inflammation. This inflammation is thought to be associated with higher rates of posterior capsular opacity, posterior synechiae, and cystoid macular edema [10–12]. Combined surgery itself can pose additional challenges in that the red reflex may be diminished for patients with vitreous hemorrhage, and if the cataract happens to be very dense, the cornea can become edematous during phacoemulsification, compromising the surgeon's view.

In 2010, Oshima et al. published the first series of cases using 27-gauge vitrectomy [13]. FLACS was started in 2009 with incorporation into routine surgical practice occurring in 2010 [4, 14]. The efficacy and safety of FLACS with 23-gauge and 25-gauge vitrectomy have been published [5, 15, 16].

In the study reported by Demetriades et al. including 122 eyes of 111 patients who underwent combined phacovitrectomy in eyes with significant cataract and coexisting vitreoretinal pathology, iris capture by intraocular lens optic was observed in 4 (5.2%) of the patients postoperatively [15]. Gomez-Resa et al. published a study including 21 eyes of 21 patients who underwent 23-gauge pars plana vitrectomy and femtosecond laser-assisted cataract surgery. The diameter of the capsulorhexis was chosen as 4.8 mm in eyes where the use of a gas tamponade was expected (e.g., macular hole) and 5 mm in all other eyes, and they reported that all the IOLs remained well positioned at 3 months follow-up [16].

Recently, in 2017, Rizzo et al. reported a study investigating the safety and efficiency of combined femtosecond laser-assisted cataract surgery and sutureless 25-gauge and 27-gauge vitrectomy. The study included 15 patients with varying vitreoretinal pathologies (macular hole, *n* = 6; epiretinal membrane, *n* = 6; vitreous hemorrhage, *n* = 2; retinal detachment, *n* = 1). The authors stated that despite the use of intraocular gas or air tamponade in patients diagnosed with macular pathologies, IOL subluxation or posterior capsule opacification occurred in none of the patients. Furthermore, the toric IOL implantation was performed in 4 of the patients and IOL rotation was unremarkable (1.64 ± 0.28 degrees) 3 months postoperatively [17]. However, there is still limited literature data available concerning the benefits and disadvantages of FLACS with 27-gauge vitrectomy. Therefore, we published the current study.

Lasers can currently support or replace various aspects of cataract surgery including the creation of clear corneal incisions (CCI), the creation of the capsulotomy, and the fragmentation of the lens nucleus. Accurate docking is vital for the success of the FLACS procedure since poor or inadequate docking can affect all aspects of the laser process [18]. Retrobulbar or peribulbar anesthesia can result in chemosis and can impede cone placement and may result in docking loss during femtosecond laser operation. Therefore, we applied the femtosecond laser under topical anesthesia, and the rest of the operation was performed under retrobulbar block anesthesia. Studies have found that clear corneal incisions created with femtosecond lasers are square and significantly more resistant to deformation and leakage compared with manually created incisions [19, 20]. However, current practice requires a partial-thickness incision, which is then completed manually as soon as the patient is sterile. Therefore, we think that avoiding the breaching of the anterior chamber before the patient is sterile may impact on the perceived advantages of laser incisions. Also, Kelkar et al. have reported recommending manual corneal incisions due to significant stromal hydration and surgical visualization difficulty intraoperatively in one patient where the incision was placed more centrally [21]. For this reason, we prefer to perform manual corneal incisions in FLACS-assisted combined surgeries.

Achieving the best functional and anatomical outcomes is highly dependent on successful intraocular lens implantation in combined vitreoretinal surgery. Pupillary capture, tilt, or decentralization of the intraocular lens may induce astigmatism and high-order aberrations that lead to distortion and decreased visual acuity [22]. Combined vitreoretinal surgery and intravitreal tamponades may induce IOL tilt, decentralization, and pupillary capture [6, 23]. These complications can be decreased by creating a smaller capsulorhexis [23]. Creating a more consistent and precisely sized and shaped capsulotomy appears to be a significant advantage of FLACS [24, 25]. The literature shows that femtosecond laser-generated capsulotomies are invariably more precise than manual capsulorhexis, resulting in better centration and more uniform IOL optic overlap [26, 27]. In the current study, we created a 4.9 mm capsulotomy diameter that covers a 6 mm optical area of the IOL with the femtosecond laser. All IOLs remained stable inside the capsular bag during the operations and the postoperative follow-up. Also, comparison of preoperative target spherical refractive errors and postoperative spherical equivalent refractive errors at 6 months showed no statistically significant difference. We believe that the ideal position of the IOL within the capsular bag contributes to good refractive results in combined surgery with intraocular tamponades.

Nucleus fragmentation reduces the energy and time of phacoemulsification [5, 28]. Palanker et al. reported a 39% decrease in energy using the phacoemulsification system [29]. Conrad-Hengerer et al. reported similar results [30]. Nagy et al. reported that the femtosecond laser decreased phacoemulsification operative time by 51% in a porcine eye study [4]. Benefits of decreased energy and time of phacoemulsification include reduced central corneal endothelial cell loss, corneal edema, and inflammation during the postoperative period [31, 32]. In the current study, the mean time of the phacoemulsification and intraocular lens implantation was 14.65 ± 0.93 minutes and the mean total surgical time was 50.31 ± 9.01 minutes. All patients had clear corneas during vitreoretinal surgery, and there was no posterior synechiae, fibrin reaction, capsular opacification, or pseudophakic cystoid macular edema postoperatively. We believe that femtosecond laser cataract surgery has the potential to affect all these factors positively with benefits of decreased time and energy in combined surgery.

FLACS showed excellent results in cataract surgery, but still there are many complications to be considered such as suction problems, conjunctival hemorrhage, capsular tears, miosis, and endothelial damage. Conjunctival hemorrhage and miosis are seen mostly among these complications, and the incidences of these are reported to be about 34% and 32%, respectively, in FLACS [33]. Kelkar et al. reported 4% miosis in their series, which was overcome by use of intraoperative intracameral adrenaline (0.001%), and the low incidence of miosis may be related with use of preoperative topical nonsteroidal anti-inflammatory drug and in addition added topical tropicamide plus phenylephrine eye drop immediately after laser treatment [21]. In the current study, miosis was not detected in any patient and we believe this to be related with use of preoperative topical nonsteroidal anti-inflammatory drug and added topical dilatation eye drop immediately after femtosecond laser treatment. Also, although two patients had conjunctival hemorrhage related with docking, there were no other complications of FLACS. On the other hand, the limitations of this technique include high financial requirement and the need to shift the patient to another table or room for the other surgical procedures. A potential advantage of combined surgery is preventing the patient from undergoing a further operation with high costs, anesthesia, and surgical risks. However, there is great dilemma regarding the high cost of FLACS in combined surgery. Despite the high cost of FLACS, we believe that FLACS is useful in combined surgery. The main benefits of FLACS in combined surgery with intraocular tamponades are desirable insertion of the IOL in the bag with regularly shaped capsulotomy. In our clinic, we transfer patients to another room after the laser procedure. This can result in increased time spent by the surgeon, but it is negligible compared to the potential advantages of FLACS such as regular capsulotomy and decreased energy and time of phacoemulsification.

Although the current study is the largest series with FLACS and 27-gauge vitrectomy with gas tamponade, it contains some limitations. It does not give an idea about the effect of FLACS on endothelial damage or creation of corneal incision. It has a relatively small number of cases and no control group with conventional cataract surgery. Also, this study does not give an idea about FLACS and 27-gauge vitrectomy with silicon oil.

In summary, despite the high cost of FLACS, it appears to be safe in 27-gauge combined vitrectomy surgery. It offers perfect capsulotomy and nucleus fragmentation even in the absence of red fundus reflex and precise IOL stability during air-fluid exchange and postoperative period with intraocular gas tamponade. In future, FLACS and 27-gauge sutureless combined surgery may be a future trend in appropriate cases.

## Figures and Tables

**Table 1:** Exclusion criteria.

(1)	Any previous intraocular surgery
(2)	Traumatic cataract
(3)	Pseudoexfoliation syndrome
(4)	Total or partial absence of the iris
(5)	Phacodonesis or lens subluxation
(6)	Posterior or anterior synechiae
(7)	Corneal opacification preventing adequate anterior segment visualization
(8)	Intraocular pressure >21 mmHg on the day of surgery
(9)	Inability to follow verbal instructions (mental or hearing alterations), claustrophobia, physical tremor, or limitation for remaining in supine position during the laser procedure
(10)	Not accepting participation in the study
(11)	Follow-up under 6 months
(12)	Incomplete medical records

## Data Availability

The data used to support the findings of this study are available from the corresponding author upon request.
